# Editorial: Synthetic Microbial Ecology

**DOI:** 10.3389/fmicb.2021.757848

**Published:** 2021-11-11

**Authors:** Thomas Brüls, Franz Baumdicker, Hauke Smidt

**Affiliations:** ^1^Génomique Métabolique, Genoscope, Institut François Jacob, CEA, CNRS, Univ Evry, Université Paris-Saclay, Evry, France; ^2^Cluster of Excellence “Controlling Microbes to Fight Infections”, University of Tübingen, Tübingen, Germany; ^3^Department of Mathematical Stochastics, University of Freiburg, Freiburg, Germany; ^4^Wageningen University & Research, Wageningen, Netherlands

**Keywords:** synthetic microbial ecology, microbial communities, mathematical modeling, microbiome, microbial interactions, metagenomics

Richard Feynman's inevitable quote “What I cannot create, I do not understand” was not only adopted by synthetic biologists engineering individual molecular machines or rewiring cell circuitry, but also by synthetic ecologists seeking to manipulate whole microbial communities for dedicated purposes. The present issue themed on synthetic microbial ecology features contributions illustrating on-going research in this very active and vivid field.

Synthetic microbial ecology seeks a holistic, dynamic, and mechanistic understanding of microbiomes viewed as interactive and organized biological systems. However, the lack of mechanistic understanding of trophic, metabolic, and ecological interactions is currently the main obstacle to the engineering of microbiomes, ultimately preventing the restoration of compromised systems. Community genomics (metagenomics) has allowed the identification of numerous microorganisms of interest, but omics data alone is limited in its ability to extensively probe complex ecological interactions, even if we know empirically that dynamic microbial communities are key to environmental, biotechnological, and biomedical processes.

Synthetic microbial ecosystems are model systems (i.e., amenable to experimentation and modeling) of reduced and known complexity used to investigate the organization and stability of communities; these have the power to identify generic quantitative patterns and to measure the relative importance of stochasticity in community operation. Ultimately, the integration of *in vitro* experiments with mathematical modeling will both test our understanding and enable applications. This endeavor can typically be carried out in a bottom-up or top-down fashion, with the former focusing on the identification of conditions necessary to generate specific interaction patterns and dissecting competition and cooperation relations, while the latter focuses on overall functions and the resilience of microbial systems.

Estrela et al. contribute a stimulating review stressing the value and benefits of enrichment communities, an inherently top-down approach. The authors note first that recent advances in robot-assisted culturing platforms and sequencing technologies allow to quantitatively track the assembly process of enrichment communities in high-throughput. Therefore, by monitoring such community assembly experiments in large numbers of synthetic habitats, where extrinsic sources of variation among replicates can be controlled, the reproducibility and predictability of community assembly can be investigated at different organizational levels, together with their relationship to metabolic and ecological drivers.

The authors make a strong case for top-down approaches, reminding that the difficulties of controlling natural habitats, together with our incomplete understanding of the forces at play there, constitute almost insurmountable obstacles to theory development. However, many of these challenges can be circumvented in enrichment communities, consisting of natural microbiomes cultivated *ex situ* under well-controlled conditions. Thus, unlike communities engineered from the bottom-up, enrichment studies can avoid often problematic assumptions about the relevant factors (e.g., trophic interactions and/or phenotypes) at play. The various forces driving community assembly can be rationalized into mathematical models that can be evaluated by their success at explaining and predicting the abundance of species and their variability.

The authors mention some of their own findings, for example that the repetition of the same enrichment experiment (i.e., with the same inoculum) frequently yields communities differing at the level of their species composition, yet that are strongly convergent at higher taxonomic levels, thus potentially reflecting an underlying metabolic structure. On the mathematical level, the authors have extended the classical resource competition model of MacArthur-Levins to include metabolic cross-feeding. This is a fundamental step as the productivity of many communities appears co-limited by multiple nutrients simultaneously, and entails the “diversity begets diversity” principle observed in community assembly, as more niches may be created by cross-feeding as more species are added to an initial species-poor environment. The authors make a convincing point that understanding the fascinating question of how co-limitation affects biodiversity could be addressed using enrichment communities. Relevant to bottom-up approaches, the authors also mention a simple rule (not necessarily observed in generalized Lotka-Volterra models) having a strong predictive value in small communities, and stating that in a multiple species mixed culture, only the subset of species that all coexist with one another in pairwise co-culture will survive, whereas those that are excluded by any member of that subset will go extinct.

Gorter et al. report an exploratory study on the evolution of a type of competitive interaction known as interference competition. Contrary to exploitative competition, where interactions among individuals are indirect (e.g., mediated solely through a common resource's availability), in interference competition negative interactions occur directly via antagonistic traits (e.g., the production of toxic chemicals). Interference competition contributes also to positive frequency-dependent interactions, and can drive the structuring of natural communities that way. Importantly, interference competition is modulable and involves a trade-off between the cost of producing the antagonistic trait and the benefits derived from it. In structured environments, producers of toxins inhibiting closely related competitors have demonstrated advantages, but under well-mixed conditions, cheaters who don't endure the cost of production reap the benefits because the toxins are shared among individuals.

The authors explored if antagonism could evolve by selection of a pyocin producing strain in a spatially structured environment for 10 serial transfers in the absence or presence of one of three non-evolving “recipient” strains. They assayed the evolved populations for fitness and for their capacity to inhibit the growth of recipient strains relative to the ancestral strain that founded the evolution experiment. They noticed that all strains, including those evolved in the absence of recipients, had higher fitness in recipient environments than in environments without recipient, suggesting some adaptation to the conditions of culture and to the presence of recipients. On the other hand, inhibition decreased in the absence of a recipient, while in the presence of a recipient, antagonism evolved in a way depending on the recipient used. Complementing their phenotypic characterization, the authors also sequenced selected evolved populations to assess modifications at the genomic level. Overall, these populations did not differ in easily interpretable ways, e.g., at the level of genes involved in bacteriocin production. Of notice, in addition to multiple mutations shared at the gene level, evolved strains with varying inhibition levels acquired novel prophage elements not present in the ancestral strain, reminiscent of previous accounts of a possible advantage of harboring lysogenic prophages.

Zandbergen et al. report on interactions between the urinary microbiota and uropathogens. Among the numerous populations of microorganisms living in various human body niches, the urinary tract has been understudied. This is primarily because it was considered sterile in the absence of infection, although modern sequencing and enhanced culturing techniques have upended this paradigm by revealing the presence of an autochthonous microbiome (with more than 100 different species isolated so far). The role of the healthy urinary microbiome in maintaining bladder homeostasis and preventing urinary tract infection is an emerging field nowadays. In particular, the mutual interactions between uropathogens and the resident microbiota are largely unknown, even if the gastrointestinal tract is commonly considered to be the origin of most bacterial infections in the urinary tract.

The authors investigated interactions between uropathogens, isolated from elderly individuals suffering from urinary tract infections, and bacteria isolated from the urinary tract of asymptomatic individuals, using growth measurements in conditioned artificial urine media. In relation to the previous report of Gorter et al., bacteria grown in conditioned medium can be viewed as acceptors, whereas bacteria from which these synthetic media were generated from are the donors. These experiments led the authors to note that uropathogens and commensal bacteria can affect each other's growth, constituting an early step in elucidating the role of microbial interactions in urinary microbial ecosystems. Metagenomics could be most useful in such bottom-up experiments in order to assess and cross-link the compositions of communities “in a bottle” with those thriving in natural *in situ* systems. Wisely, the authors acknowledge that the host may play an important role in the potential pathogenicity of uropathogens [as suggested by early human metagenome-wide association studies (MGWAS) that highlighted probable roles for components of immune pathways].

Soil is a complex and heterogeneous matrix, and the mechanisms used by edible fungi like morels to acquire nutrients from soil environments in order to drive their fructification remain elusive. The study of Tan et al. relied on a semi-synthetic substratum of quartz particles as an inorganic matrix, mixed with fermented compost to mimic the role of organic matter and microbiota in the soil. Compared with ordinary soils, this semi-synthetic system is more controllable and reproducible in many abiotic and biotic factors. Overall, microbiota successions, substrate transformation, and the activity level of key enzymes were compared between three types of substrata associated to very different yields of morel fruiting bodies. The authors used these complementary measurements to compare the patterns between the different semi-synthetic substrata with respect to key ecophysiological factors driving the fructification process. This allowed them to identify a substratum featuring microbiota-driven advantages in accumulating lipids and in establishing a balance between nitrogenous compounds (nitrate vs. ammonia).

Last but not least, Del Frari and Ferreira ponder on the fact that numerous definitions have been proposed for the term “microbiome,” which is widely used in the literature nowadays, and that its meaning has shifted from organisms viewed as taxonomic units (microbiota) to their collective genetic material (pangenomes). The authors monitored the use of terms for cultivability, taxonomic identification, microbial multiplicity (i.e., the number of organisms involved) and assemblage reproducibility in ~100 peer-reviewed microbiome articles, leading them to propose the alternative function-centric term “skopobiota” (“skopos” means “purpose” in old greek). In this context, a skopobiota refers to microbial assemblages that, unlike microbiomes, are purpose built to study the behavior of system components or to carry out well-defined tasks (e.g., biotechnological control). Nevertheless, the authors acknowledge that inaccuracy might be more fundamentally rooted in the nature of knowledge rather than in terms, which may not necessarily be a bad thing if we remind of Feynman's (among others) remark to look at problems from several points of view when the physical situation at hand can not be analyzed directly (e.g., by solving differential equations).

In conclusion, even though truly transformative applications of synthetic microbial ecology are foreseeable, massive knowledge gaps hinder our understanding of the dynamics, stability, and function of simplified ecosystems. For example, we are currently unable to precisely predict the auto-structuration of communities emerging from relatively long term experiments, like those described by Estrela et al. where growth on minimal media with glucose as the single carbon source led to a structuring of the community into two groups implementing distinct energy metabolisms, leading to a coupling of fermentative vs. respiration-based lifestyles. The investigation of reduced model systems, as reported in this thematic issue and derived from either top-down or bottom-up approaches, together with close integration of *in vitro* experiments with mathematical modeling should deepen our understanding of ecological, metabolic, and trophic interactions underpinning these microbial assemblages and their actionability. Targeting such “known unknowns” seems the way to go, most probably uncovering “unknown unknowns” along the road.

## Author Contributions

TB drafted the manuscript and all authors contributed substantially to the final version and approved it for publication.

## Conflict of Interest

The authors declare that the research was conducted in the absence of any commercial or financial relationships that could be construed as a potential conflict of interest.

## Publisher's Note

All claims expressed in this article are solely those of the authors and do not necessarily represent those of their affiliated organizations, or those of the publisher, the editors and the reviewers. Any product that may be evaluated in this article, or claim that may be made by its manufacturer, is not guaranteed or endorsed by the publisher.

